# Calling by Concluding Sentinels: Coordinating Cooperation or Revealing Risk?

**DOI:** 10.1371/journal.pone.0025010

**Published:** 2011-10-03

**Authors:** Linda I. Hollén, Matthew B. V. Bell, Alexis Russell, Fraser Niven, Amanda R. Ridley, Andrew N. Radford

**Affiliations:** 1 School of Biological Sciences, University of Bristol, Bristol, United Kingdom; 2 Department of Zoology, University of Cambridge, Cambridge, United Kingdom; 3 Pied Babbler Research Project, Kuruman River Reserve, Vanzylsrus, Northern Cape, South Africa; 4 Centre of Excellence, Percy FitzPatrick Institute, University of Cape Town, Rondebosch, South Africa; Georgia State University, United States of America

## Abstract

Efficient cooperation requires effective coordination of individual contributions to the cooperative behaviour. Most social birds and mammals involved in cooperation produce a range of vocalisations, which may be important in regulating both individual contributions and the combined group effort. Here we investigate the role of a specific call in regulating cooperative sentinel behaviour in pied babblers (*Turdoides bicolor*). ‘Fast-rate chuck’ calls are often given by sentinels as they finish guard bouts and may potentially coordinate the rotation of individuals as sentinels, minimising time without a sentinel, or may signal the presence or absence of predators, regulating the onset of the subsequent sentinel bout. We ask (i) when fast-rate chuck calls are given and (ii) what effect they have on the interval between sentinel bouts. Contrary to expectation, we find little evidence that these calls are involved in regulating the pied babbler sentinel system: observations revealed that their utterance is influenced only marginally by wind conditions and not at all by habitat, while observations and experimental playback showed that the giving of these calls has no effect on inter-bout interval. We conclude that pied babblers do not seem to call at the end of a sentinel bout to maximise the efficiency of this cooperative act, but may use vocalisations at this stage to influence more individually driven behaviours.

## Introduction

Sentinel systems, where individuals of social species adopt raised positions to scan for danger [1], are associated with substantial anti-predator and foraging benefits to group members [2], [3], [4], [5], [6], [7]. These systems represent a sophisticated form of cooperation and involve constant information exchange between groupmates. It is well known that sentinels provide vocal information to foragers about potential or actual predation risk [4], [6], [7], and that both parties use vocalisations to convey information about their state and likely contributions to sentinel behaviour [4], [8], [9]. While it has also been suggested that information exchange is used to maximise the efficiency of the system [4], [6], [10], this aspect of sentinel behaviour is the most poorly understood and most speculative. We have good evidence that the watchman's song (quiet vocalisations produced throughout a sentinel bout; [11]) acts to advertise sentinel presence, amplifying the benefits of having a sentinel [4], [5], [10]. However, it is unclear whether sentinel bouts are actively coordinated (but see [4]) or the likelihood of sentinel replacement is adjusted to the level of risk. Determining the mechanisms underpinning cooperative acts is important if we are to understand fully the evolution and maintenance of cooperation.

A candidate mechanism for maximising the efficiency of the sentinel system involves the particular vocalisations given by sentinels of some species prior to coming down or during their descent to the ground [12] (cited in [2], [13]). One possibility is that these calls aid in coordinating cooperation by conspicuously advertising the absence of a sentinel and the need for replacement. Mechanisms ensuring effective coordination of individual bouts would reduce the time each individual spent foraging unprotected, and a vocal signal would minimise the disruption to foraging caused by visual monitoring of sentinel presence (see [4], [5], [10]). Another, non-mutually exclusive, possibility is that calls given at the end of a bout act as a signal of risk, advertising the urgency with which replacement is needed. Several studies have demonstrated that the level of urgency can be conveyed in alarm calls (e.g. [14], [15], [16]), and recent work on meerkats (*Suricata suricatta*) suggests that particular close calls given just after an individual has scanned for predators function as an “all clear” signal, informing receivers that the likelihood of encountering a predator is relatively low and leading to a subsequent reduction in forager vigilance [17].

**Figure 1:**
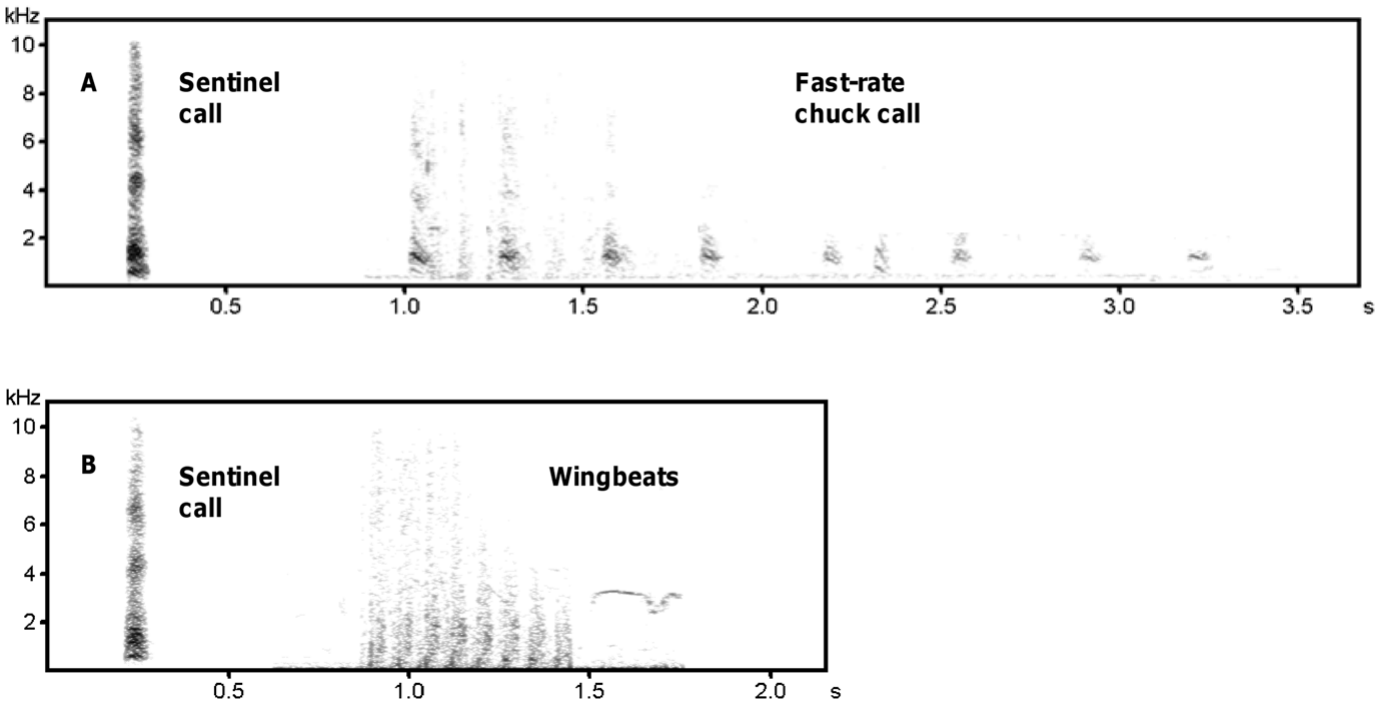
Illustrative spectrograms of the two different playback treatments. In one treatment (A), sentinel calls (the watchman's song) were followed by the fast-rate chuck call; in the other treatment (B), sentinel calls were followed by the sound of wingbeats from a descending sentinel. Spectrograms only show the last section of each playback treatment and not the preceding 2 min of sentinel calling (see Methods).

Pied babblers (*Turdoides bicolor*) have a well-studied sentinel system which offers the ideal opportunity to investigate the potential function of calls given at the end of guarding bouts. Pied babblers are cooperatively breeding birds of arid Southern Africa, subject to intense predation from numerous avian and terrestrial predators. The birds forage on the ground, where individual vigilance is restricted [18], [19], and sentinels produce vocalisations which appear to maximise the efficiency of the system. In particular, sentinels inform foragers about their presence, position and the current level of risk by giving a continuous watchman's song, allowing foragers to adjust their behaviour appropriately [5], [6], [20]. At the end of a bout, descending sentinels also produce a fast-rate chuck call (see Fig. 1A). However, these calls are not given at the end of every bout, and their involvement in regulating sentinel changeovers and/or the speed with which it is done is unknown.

Here we use observational and experimental data to investigate the function of fast-rate chuck calls given at the end of sentinel bouts. Specifically, we ask: (i) when pied babbler sentinels give fast-rate chuck calls, and (ii) what effect these calls have on the interval between sentinel bouts? If fast-rate chuck calls signal the need for replacement to aid rotation, they should be given more often when foragers are less able to detect the absence of a sentinel, such as in dense habitats (when descending sentinels are more likely to be visually obscured) and/or high wind (when the increased background noise makes the absence of the watchman's song harder to determine) (see [21], [22], [23]). We would also expect that the experimental playback of fast-rate chuck calls should reduce the latency between individual sentinel bouts. If fast-rate chuck calls signal current risk, there are two possibilities. First, calls may indicate high predation risk at the time of descent and the crucial need for quick sentinel replacement. In such a situation, we would also predict calls to be given more often in dense habitats and/or high wind (when predators are harder to spot; e.g. [22], [23]), and bout latency to be reduced when calls are given. The other possibility is that fast-rate chuck calls act as an “all clear”, signalling low risk and no immediate need for replacement. If so, they should be more likely when sentinels can be relatively certain that there is no urgent threat, such as in open habitats and/or in conditions with little wind, and the latency between sentinel bouts should be longer when a fast-rate chuck call is given compared to when it is not.

## Methods

### Ethics Statement

This research adhered to the Association for the Study of Animal Behaviour/Animal Behavior Society Guidelines for the Use of Animals in Research, the legal requirements of the country (South Africa) in which the work was carried out and all institutional guidelines. The University of Bristol Animal Services Ethical Committee approved the procedures under UIN: UB/06/035, and the Northern Cape Conservation Authority in South Africa provided research permission to work at the Kuruman River Reserve (permit number: FAUNA 577/2010; Application ID: 6914).

Trapping, ringing and the taking of blood samples was conducted under South African Bird Ringing Unit (SAFRING) licence no. 1263 issued to Amanda Ridley. Individuals were caught using a walk-in trap, which was placed 20–50 m away from the group to minimise disturbance to other group members. Birds were enticed into the trap using mealworms as bait. Traps were never left unattended and as soon as it was triggered, the trap was covered with a dark blanket to calm the bird. All birds were removed within 5 min of capture, ringed and a blood sample (c. 50 µl) obtained by brachial venipuncture. The ringing process rarely took longer than 5 min. Trapping always occurred during the day, at least one hour after sunrise or before sunset, when birds were displaying normal foraging behaviour. Trapping never occurred at potentially stressful times, such as during inter-group interactions or predator-mobbing events. There were no adverse effects of the trapping and ringing procedure: birds were promptly released back to their group following completion of ringing and resumed normal foraging behaviour within 10 min of release; birds were not attacked by other group members on their return to the group; and no bird was injured or died during the ringing process.

Birds showed no adverse reactions to the observations or the playback experiment conducted; all studied groups were habituated to the presence of observers and had experienced playback experiments previously.

### Study Site and Species

The study site is located on the Kuruman River Reserve in the Southern Kalahari, South Africa (26°58′S, 21°49′E). The vegetation comprises a combination of annual and perennial grasses (*Eragrostis*, *Aristida*, *Schmidtia*, *Stipagrostis*) and *Acacia* and *Boscia* trees, and the average annual rainfall (measured daily at the study site) is 217 mm. See [3], [24] for further details of the climate and vegetation.

We studied eight groups of pied babblers (mean ± SE adult group size during study = 5.5±0.5, range 3–9) that were colour-ringed and habituated to allow observation from 2–3 m distance (see [18]). Adults (>12 months old) were divided into dominants (the putative breeding pair) and subordinates (all other adults); paternity analysis has confirmed that the vast majority of young (95%) are the offspring of the putative breeding pair [25]. Breeding females always incubate the eggs overnight; breeding males were identified from mid-air courtship chases and copulations with breeding females. Pied babblers are sexually monomorphic in plumage and size, so subordinates and fledglings were sexed using a DNA test (see [26] for details). Blood samples were obtained by brachial venipuncture (see ‘Ethics Statement’ and [27] for details), kept cool in the field and then stored at 4°C until DNA extraction and analysis in the laboratory.

### Observational Data

Observations were made for 4 h following dawn and for 2–3 h before dusk, between April and July 2009. Sentinels were defined as individuals perched >1 m above the ground and actively scanning for predators while other group members were foraging [5], [28]. *Ad libitum* data were collected during one-hour sessions (mean ± SE sessions per group = 16±1.6, range 10–25, n = 8 groups), when we recorded: (1) the start and end of every sentinel bout; (2) sentinel identity; (3) whether a descending sentinel gave a fast-rate chuck call; (4) habitat type; and (5) wind condition. We defined three habitat types: *grass* (ground dominated by long grass where foragers and any awaiting terrestrial predators are completely covered); *thickets* (ground covered with small bushes and thickets with open areas in between); and *open* (stretches of exposed sand with little vegetation). Wind condition was defined as *low* (occasions when there was no vegetation movement or leaves and grass were moving slightly) or *high* (occasions when tree branches were clearly moving). From (1) we extracted the latency between consecutive bouts by the same or a different group member. All data were recorded onto a Palm TX PDA (Palm Inc., Sunnyvale, CA, USA), which automatically noted the time of each event.

### Playback Experiment

To test experimentally whether fast-rate chuck calls affect the latency between consecutive bouts, the eight groups were presented with two playback trials: one involved 2 min playback of sentinel calls (the watchman's song; [5]) at a standardised rate (15 calls/min), followed by the fast-rate chuck call (see Fig. 1A); the other (control) involved 2 min playback of sentinel calls at the same standardised rate, followed by the sound made by wingbeats as a sentinel comes off guard, mimicking a sentinel descending without vocalising (see Fig. 1B). Calls and wingbeat sounds used in the experiment were recorded from the groups' dominant males. Trials to the same group were conducted on separate days (range 1–5 days between trials) and the order of trials to different groups was counterbalanced: four groups received the playback of sentinel calls+fast-rate chuck call first and the other four received the playback of sentinel calls+wingbeats first. Playbacks were of the same sound intensity as natural calls (determined using a Tandy sound-level meter), were broadcast from a Sony SRS-A35 speaker positioned on the observer's head (observer stood at the base of a tree), and were conducted when no natural sentinel had been present for at least 2 min and there had been no alarm calls for at least 10 min. The latency between sentinel bouts was recorded on a Palm TX PDA (Palm Inc., Sunnyvale, CA, USA).

### Data Analysis

All data were analysed in R for Microsoft Windows 2.12.1. [29]. Generalized Linear Mixed Models (GLMM, binomial error structure and logit link function) and Linear Mixed Models (LMM, Gaussian error structure and identity link function) were fitted using the lme4 package [30] and the ‘lmer’ function (REML fit). Mixed models allow the inclusion of both fixed and random terms, the latter accounting for repeated measures of the same individual and group. Individual identity and group identity were initially included as random terms in all models, but the former was always removed because it explained zero variance.

We used a GLMM to examine the influence of habitat type and wind condition on the likelihood of a fast-rate chuck call (YES/NO) being produced at the end of a sentinel bout, while controlling for individual sex and status (dominant or subordinate). We then used a LMM to examine how the production of a fast-rate chuck call (YES/NO) influences the latency between consecutive sentinel bouts, while controlling for habitat type, wind condition, foraging group size (used instead of total group size to account for individuals that were temporarily missing from the group) and total rainfall in mm during the previous week (see also [31]). Significance of each explanatory term within the models was examined using a classical model simplification approach with chi-square tests (log-likelihood ratio tests) measuring the change in deviance (Δdeviance). By removing each term in turn from a full model including all terms (including interactions), we established a minimal model with only significant terms remaining. The significance of these latter terms was established by removing each of them in turn from the minimal model and comparing the reduced model to the complete minimal model. The p-value of non-significant terms was established by adding each term in turn to the minimal model. Terms (main effects and interactions) were judged as adding significant explanatory value if their removal resulted in a change in deviance producing a p value<0.05. Prior to analysis, latency between sentinel bouts was square-root transformed (models applied to transformed values were better fitting than models applied to non-transformed values, as assessed by residual deviance and visual inspection of normality plots). Data from the playback experiment were analysed using a paired t-test with log-transformed values for bout latency.

## Results

**Figure 2:**
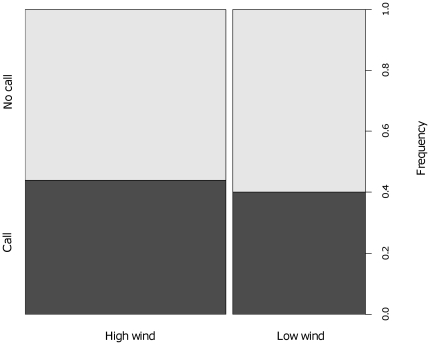
The frequency with which fast-rate chuck calls were given by different classes of sentinels. Dominant females = DF, dominant males = DM, subordinate females = SF, and subordinate males = SM. Within individual status, there was no difference between sexes (DF-DM, p = 0.12; SF-SM, p = 0.20). Within individual sex, there was a significant effect of dominance for females (DF-SF, p = 0.027, effect size = 0.207) but not males (DM-SM, p = 0.94) – dominant females produced fewer fast-rate chuck calls than subordinate females. Analysed using the package “languageR” and the function ‘pvals.fnc’ (uses Markov Chain Monte Carlo simulations; [37]).

**Figure 3:**
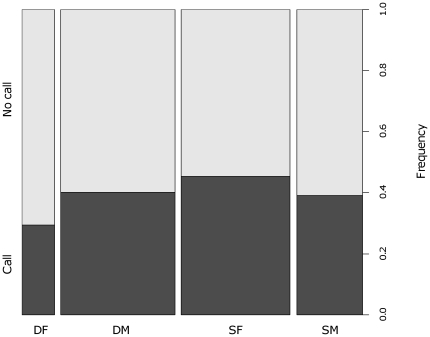
The frequency with which fast-rate chuck calls were given in conditions with high and low wind. The production of fast-rate chuck calls was statistically more frequent in high wind than in low wind.

Fast-rate chuck calls were given at the end of 44% of all sentinel bouts (n = 890 bouts by 27 individuals) with little variation between study groups (SE = ±4.8%). After controlling for a significant effect of the interaction between individual status and sex (GLMM: χ^2^ = 4.19, df = 1, p = 0.040), where dominant females produced fewer fast-rate chuck calls than subordinate females (see Fig. 2), the likelihood that a call was given was not significantly influenced by habitat type (χ^2^ = 2.77, df = 2, p = 0.25). There was a significant effect of wind condition (χ^2^ = 34.24, df = 1, p<0.001), suggesting that pied babbler sentinels were more likely to produce a fast-rate chuck call in high wind. However, the size of this effect was small (0.13±0.14) and the proportional difference between high and low wind minimal (see Fig. 3).

**Figure 4:**
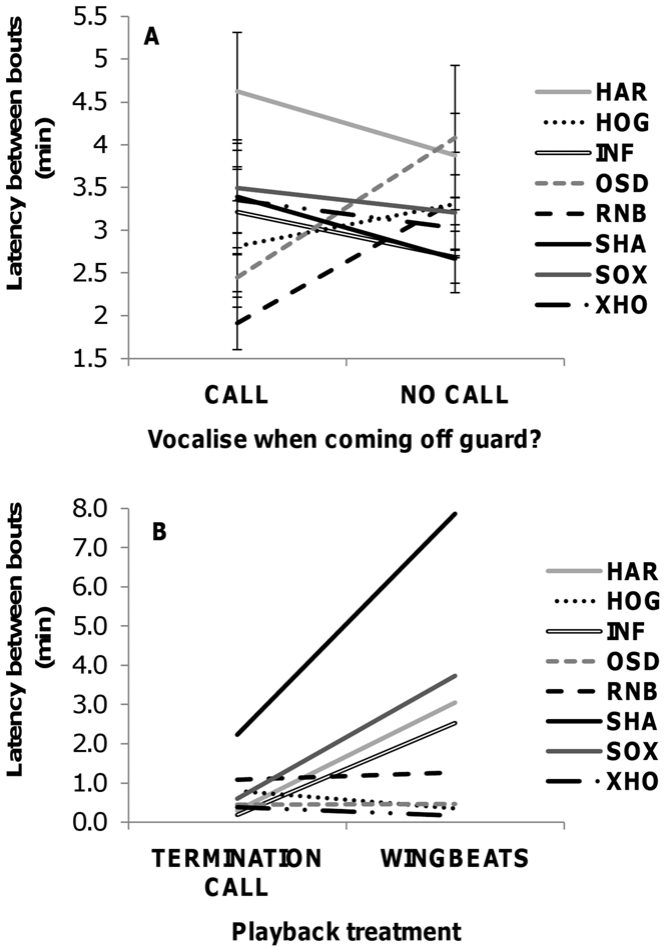
The effect of fast-rate chuck calling on bout latency. (A) Observational data showing the effect of fast-rate chuck calling vs. no calling on the latency between consecutive sentinel bouts. Shown are means (±SE) for each of the eight groups. (B) Playback results showing the effect of fast-rate chuck calls vs. the sound of wingbeats on bout latency. The three letter codes in the legend represent individual group names.

Latency between consecutive sentinel bouts ranged from 0.1 to 62.2 min (mean ± SE = 3.22±0.13 min, n = 1172 bouts by 50 individuals). We found no effect of habitat (LMM: χ^2^ = 0.24, df = 1, p = 0.89), foraging group size (χ^2^ = 0.65, df = 1, p = 0.42) nor rainfall (χ^2^ = 0.99, df = 1, p = 0.32) on bout latency. After controlling for a significant effect of wind condition, where bouts were generally initiated sooner in high wind (χ^2^ = 82.82, df = 1, p<0.001; effect size = 0.16±0.06), there was also no significant influence of calling at the end of a bout (χ^2^ = 1.24, df = 1, p = 0.27; Fig. 4A). That is, replacement speed did not differ depending on whether a finishing sentinel produced a fast-rate chuck call (mean ± SE: 3.21±0.19 min, n = 496) or not (3.24±0.18 min, n = 676). Similarly, our experimental results showed no significant difference in bout latency following playback of fast-rate chuck calls and wingbeats (paired t-test: t = −1.71, df = 7, p = 0.13; mean of difference = −0.83, 95% CI = −1.97, 0.32, power = 0.52; Fig. 4B).

## Discussion

On some occasions, a specific vocalisation is given by pied babbler sentinels as they finish a bout and descend to the ground to forage. The variation in production of this fast-rate chuck call was not related to habitat type, an ecological factor that is known to affect the likelihood of detecting group members visually and the predation risk [22], [23], and which has previously been shown to influence other sentinel decisions [31]. Although wind condition statistically influenced the production of fast-rate chuck calls, the size of the effect renders its biological relevance questionable. Moreover, the speed with which a sentinel was replaced was not affected by the giving of fast-rate chuck calls at the end of a bout. It is important to note the low experimental sample size, and thus that the power of this particular analysis alone may be inadequate to conclude reliably that there is no effect of calling. However, given that the mean difference between the experimental treatments fell well within the 95% confidence intervals and that there was also no discernable effect of calling on latency during natural observations, our data do not seem to support either of the proposed hypotheses regarding the function of these fast-rate chuck calls. That is, there is no evidence that they are used to enhance the efficiency of the pied babbler sentinel system by either aiding coordination of individual sentinels or revealing the current predation risk to foraging individuals. In this species, therefore, calls given at the end of a sentinel bout do not appear to be strongly connected with the cooperative aspects of sentinel behaviour.

Previous work has indicated that coordination of sentinels does occur to some degree in some species. A strict rotation of sentinels, where there is a regular pattern of changeovers and each individual takes over from a particular member of the group, has so far only been described for dwarf mongooses (*Helogale parvula*) [10]. In various babblers [7], [8], [13], [32], Florida scrub-jays (*Aphelocoma c. coerulescens*) [2], [33] and meerkats [3], [4], a simpler form of sentinel coordination seems to be present, whereby the number of sentinels varies little (mostly one sentinel at a time) but different individuals come and go as sentinels. A finishing sentinel is quickly replaced by another and if two individuals guard simultaneously, the initial individual in the role generally resumes foraging quickly. There is no evidence to date, however, that this coordination is assisted by calls given as a bout is terminated. In addition to our study, there is no support in Florida scrub-jays for specific calls aiding sentinel coordination by signalling the end of a bout [34]. There has been some suggestion that the intermittent watchman's song of meerkats might be used in this regard [4], but whether the continuous watchman's song of pied babblers [5] could also be used for the coordination of the sentinel system requires experimental testing.

Although several studies have shown that other vocal signals can indicate level of risk [6], [15], [16], [17], we have no strong evidence that pied babbler fast-rate chuck calls function in this regard. Unlike the guarding close calls of meerkats, which are given when a forager has recently scanned for danger and not detected any imminent threats [17], pied babbler fast-rate chuck calls do not appear to act as an ‘all clear’ signal. We did consider only the replacement speed of sentinels, though, and future studies might merit from investigating additional response variables, such as forager vigilance and group spread, which are known to be influenced by other sentinel vocalisations produced by both conspecifics [5], [6], [20] and heterospecifics [35]. However, pied babbler sentinels change the rate and pitch of their watchman's song in response to predation risk, and foragers are known to adjust their vigilance in response to these changes [6]. It is therefore possible that, in this species at least, additional signalling about current risk via a call given just at the end of a bout is rendered unnecessary.

If fast-rate chuck calls are not used to coordinate cooperation or to reveal risk, what might be their function? One possibility is that they are used in a competitive manner. When individuals act as a sentinel, they forego foraging and are not fed by others. Consequently, their hunger and the benefit of foraging will increase over time [9]. To reduce time spent searching for good foraging patches at the end of a bout, hungry sentinels may therefore use their raised position to monitor the behaviour of foragers and scrounge from those behaving in a way that indicates a good quality foraging patch (Radford et al., unpublished data). The production of fast-rate chuck calls as they descend may then act as a competitive deterrent to the patch holder, perhaps enhancing the likelihood of the sentinel securing the patch. Another possible function of these calls may be for a sentinel to announce their location on rejoining the foraging group and potentially to influence the direction of group movement (see [36]). These possibilities require experimental exploration in the future.

Although cooperative behaviours are often mediated by information exchange in general and vocalisations in particular, calling by pied babblers at the end of a guarding bout does not seem to function to maximise the efficiency of their sentinel system. It remains to be established why dominant females are less likely than other group members to produce fast-rate chuck calls, but this effect may suggest that some of the mechanisms involved in coordinating cooperative behaviours are not always adapted to maximise the efficiency of the group. Moreover, it is possible that these calls, although given at the end of a cooperative act, are instead related to the beginning of foraging, a more individually related behaviour. How social species balance the use of vocalisations for cooperative and selfish reasons is a topic that warrants future consideration.
